# Dermatological Findings in Glaucoma Patients: Comparison Between Pseudoexfoliative and Primary Open-angle Glaucoma

**DOI:** 10.18502/jovr.v17i4.12298

**Published:** 2022-11-29

**Authors:** Farnaz Ahmadpour, Yalda Nahidi, Ramin Daneshvar

**Affiliations:** ^1^Department of Dermatology, Imam Reza Hospital, Mashhad University of Medical Sciences, Mashhad, Iran; ^2^Eye Research Center, Mashhad University of Medical Sciences, Mashhad, Iran; ^3^Department of Ophthalmology, College of Medicine, University of Florida, Gainesville, FL, USA; ^5^https://orcid.org/0000-0001-8853-1348; ^6^https://orcid.org/0000-0002-0884-0907

**Keywords:** Dermatitis, Dermatologic Finding, Glaucoma, Open-angle Glaucoma, Pseudoexfoliation

## Abstract

**Purpose:**

To compare the frequency of dermatological manifestations between patients with pseudoexfoliative glaucoma and those with primary open-angle glaucoma.

**Methods:**

A cross-sectional study was done on all consecutive pseudoexfoliative glaucoma (PEXG) and primary open-angle glaucoma (POAG) patients evaluated in a tertiary eye hospital during the study period. Eligible patients were referred to the dermatology department for complete skin, hair, nail, and mucosal examinations.

**Results:**

Twenty-one patients in the PEXG group and 26 patients in the POAG group were included in this study. The most common skin manifestations in the study were seborrheic dermatitis, dry skin, and cherry angioma. The frequency of lentigines was significantly higher in the PEXG patients than in the POAG group (*P *= 0.013). More than half of the study population had seborrheic dermatitis (57.1% and 61.5% in the PEXG and POAG groups, respectively); however, the difference between the groups was not statistically significant (*P *= 0.775). Similarly, the frequencies of skin dryness, cherry angioma, nevus, psoriasis, contact dermatitis, itching, seborrheic keratoses, notalgia paresthetica, and vitiligo in the two groups were not statistically significantly different (*P* > 0.1 for all comparisons). There was no significant association between the frequency of the investigated skin manifestations and patients' age, visual acuity, intraocular pressure, and cup-to-disc ratio.

**Conclusion:**

Integumentary system disorders are pervasive in glaucoma patients, and dermatologic evaluation in glaucoma patients should be considered for diagnostic and therapeutic purposes.

##  INTRODUCTION

Pseudoexfoliation syndrome (PEXS) is a systemic condition. Many organ involvements, including lungs, heart, liver, gallbladder, kidneys, blood vessels, meninges, ear, optic nerves, eye, and eyelid skin have been reported in this syndrome.^[1,2,3,4,5]^ Histologic studies confirmed the presence of pseudoexfoliative materials in various tissues; nevertheless, the exact composition of these materials and their pathogenesis are still unknown. In ocular structures, these materials manifest as a group of proteins in the form of granular shells that resemble dandruff.^[6,7]^ Build-up of these materials within the eye can result in pseudoexfoliative glaucoma (PEXG), the most common secondary type of open-angle glaucoma.^[8]^ Similar to the situation in the eye, there is evidence that pseudoexfoliative substances are also present in large areas of the skin and viscera.^[3,9,10]^ However, there is still a lack of sufficient data about the characteristics of the manifestations of PEXS in the skin.

On the other hand, primary open-angle glaucoma (POAG) is the most common type of glaucoma in general worldwide.^[11]^ Treatment with topical eye drops, which is usually the first line of management for glaucoma patients, can cause a wide range of systemic adverse effects, including disseminated skin eruptions.^[12]^ Interestingly, skin involvement is among the most common comorbidities in all forms of glaucoma.^[13]^ However, dermatologic disease and adverse skin reactions caused by eye drops used in treating glaucoma have not been described sufficiently.^[14]^ Medical management of POAG and PEXG is usually the same, and one should expect similar drug-related skin manifestations in these two groups. As the pseudoexfoliative substance in ocular structures has also been found in skin, it seems reasonable to expect some additional clinical manifestations on dermatological evaluation of PEXG patients. To the best of our knowledge, no study has been published specifically on the clinical dermatological manifestations of patients with PEXS.

The primary purpose of this cross-sectional study was to describe clinical findings in the dermatologic evaluation of patients with PEXG and to compare them with those patients with POAG. Our working hypothesis was that the prevalence of specific dermatological manifestations that occur in the PEXG group might differ from those that occur in the POAG group. As the medical management is the same between PEXG and POAG, any variations discovered in dermatologic findings may highlight some probable associations with underlying pathology. Furthermore, as PEXG is highly prevalent in the elderly population in Iran,^[15]^ research on its multiple clinical manifestations seems relevant. We can better understand this syndrome and its various clinical presentations by identifying skin manifestations of PEXS patients. This discovered correlation can also help with timely diagnosis and management of the associated glaucoma.

##  METHODS

This was a cross-sectional study in the Ophthalmology and Dermatology Departments of Mashhad University of Medical Sciences (MUMS), Mashhad, Iran, between April and September 2019. Consecutive patients with a clinical diagnosis of PEXG or POAG were referred from the Glaucoma Clinic of Khatam Anbia Eye Hospital to the Dermatology Clinic of Emam Reza General Hospital for a thorough dermatologic evaluation. The study adhered to the tenets of the Declaration of Helsinki, and all included subjects provided written informed consent before participation. The Medical Ethics Committee at MUMS approved the study protocol (IR.MUMS.fm.REC.1394.646).

All glaucoma diagnoses were made by a single glaucoma-fellowship-trained ophthalmologist (RD). The diagnosis of glaucoma was based on characteristic optic nerve damage and/or visual field defect in the presence of high intraocular pressure (IOP) or a normal IOP with a history of either IOP-lowering medications, laser procedures, or surgeries. The PEXG was diagnosed based on the presence of glaucoma with any of the following findings: pseudoexfoliative material at the edge of the pupil and/or anterior capsule of the lens after complete mydriasis, atrophy of the pupillary sphincter, Zentmeyer line on the anterior lens capsule, or Sampaolesi line in gonioscopy. In contrast, those with POAG had a wide-open angle with normal angle pigmentation on non-indentation gonioscopy and without any finding of or underlying cause for secondary glaucoma. Exclusion criteria comprised any possible secondary causes of glaucoma, including pseudoexfoliation, history of using steroid medications or Cushing's syndrome at any time, history of trauma to the eye, history of retinal vascular disease, history of radiotherapy, history of uveitis, and history of intraocular surgery. Also, we excluded patients with skin conditions known as direct therapeutic side effects, such as allergic contact dermatitis, due to glaucoma drops.

Patients were referred to Imam Reza General Hospital for a complete skin examination. At the Dermatology Clinic, all subjects underwent a thorough dermatologic evaluation, including a complete examination of the skin, hair, nails, and mucosa by one expert dermatologist (YN). If needed, a skin smear or biopsy was used to confirm the diagnosis of cutaneous disease. All findings were recorded with a particular focus on the frequency and types of exfoliative manifestations (such as psoriasis and seborrheic dermatitis) in skin, hair, nails, and mucosa.

Central tendency measures were used to summarize the quantitative variables. Normal distribution of data was examined using the Shapiro–Wilk test and normality plots, and based on that, either the student *t*-test or Mann–Whitney U test was used to compare the two groups. Qualitative data was presented by their frequencies and percentages and compared between the groups using the chi-square test. All statistical analyses were performed with SPSS software (Version 18, IBM SPSS Statistics, IBM Corporation, Chicago, IL, USA). Statistical significance was set at a *P* < 0.05 level, and the significance level was adjusted for multiple comparisons with Bonferroni correction as appropriate.

##  RESULTS

In this study, a total of 47 subjects were enrolled, including 21 PEXG and 26 POAG cases. Seven patients in the PEXG group and 12 in the POAG group were females (*P *= 0.551). Patients with POAG were younger than those with PEXG (58.4 ± 15.2 vs 66.7 ± 8.3 years, *P *= 0.023). More POAG patients were in the range of 30–50 years (8 vs 2 in PEXG); however, the age distribution was almost similar for the 51–70 years and 71–90 years age groups. There was no significant difference in visual acuity (VA), IOP, and cup:disc ratio (CDR) between the two groups [Table 1].

**Table 1:** Baseline clinical characteristics of patients included in the study.


		**PEXG**	**POAG**	***P*** -value*
Number of subjects		21	26	
Male: Female		14:7	14:12	0.551
Age, yr (Mean ± SD)		66.7 ± 8.3	58.4 ± 15.2	0.023
Age groups (yr)	30–50	2	8	0.150
	51–70	9	11	0.970
	71–90	10	7	0.146
VA, logMAR (Mean ± SD)	OD	0.28 ± 0.29	0.21 ± 0.22	0.466
	OS	0.18 ± 0.23	0.22 ± 0.23	0.452
IOP, mmHg (Mean ± SD)	OD	15.7 ± 6.0	15.2 ± 4.1	0.838
	OS	16.0 ± 6.1	14.2 ± 2.3	0.488
CDR (Mean ± SD)	OD	0.72 ± 0.19	0.66 ± 0.22	0.330
	OS	0.74 ± 0.22	0.67 ± 0.23	0.240
LP or NLP eyes		5	2	0.217

OD, oculus dexter (right eye); OS, oculus sinister (left eye); CDR, cup: disc ratio; IOP, intraocular pressure; PEXG, pseudoexfoliative glaucoma; POAG, primary open-angle glaucoma; LP, light perception; NLP, no light perception; logMAR, logarithm of minimum angle of resolution; SD, standard deviation ${}^{*}$ Significant *P*-value with Bonferroni adjustment is < 0.004				

**Table 2:** Frequency of dermatologic manifestations in the study groups.


	**PEXG (** ***n*** ** = 21)**	**POAG (** ***n*** ** = 26)**	***P*** -value*
Seborrheic dermatitis	12 (57.1%)	16 (61.5%)	0.775
Dry skin	11 (52.4%)	7 (26.9%)	0.130
Cherry angioma	8 (38.1%)	9 (34.6%)	0.990
Nevus	1 (4.8%)	3 (11.5%)	0.617
Psoriasis	1 (4.8%)	1 (3.8%)	0.990
Seborrheic keratosis	2 (9.5%)	1 (3.8%)	0.579
Lentigo	5 (23.8)	0 (0%)	0.013
Dermatitis	2 (9.5%)	1 (3.8%)	0.579
Pruritis	3 (14.3%)	4 (15.4%)	0.990
Vitiligo	0 (0%)	2 (7.7%)	0.495
Notalgia paresthetica	1 (4.8%)	1 (3.8%)	0.990

PEXG, pseudoexfoliative glaucoma; POAG, primary open-angle glaucoma ${}^{*}$ Significant *P*-value with Bonferroni adjustment is < 0.005			

All PEXG patients and 24 (92.3%) POAG patients had at least one abnormal dermatological finding (*P *= 0.495). The most common dermatological manifestations in patients with PEXG were seborrheic dermatitis, dry skin, and cherry angioma. Other dermatologic manifestations of these patients included idiopathic guttate, hypomelanosis on the trunk and limbs, xeroderma pigmentosome and severe aging, lentigo maligna, and cafe au lait macules. In those with POAG, the most common dermatological findings were seborrheic dermatitis, cherry angioma, and dry skin. Other specific manifestations seen in these patients included androgenic alopecia, telogen effluvium due to acetazolamide, nail-biting, pityrosporum folliculitis, sebaceous hyperplasia, nail fungal infection, alopecia areata, photosensitivity, macular amyloidosis, lipoma, and venous lake. Table 2 represents the frequency of different dermatologic manifestations in the study participants.

Moreover, 12 patients with PEXG and 16 with POAG had seborrheic dermatitis (*P *= 0.775). One patient in the PEXG group and two in the POAG group had seborrheic findings on both head and face.

Eleven patients in the PEXG group and seven in the POAG group had dry skin manifestations, mainly in their extremities. Although the frequency of dry skin was almost twice in those with PEXG compared to the POAG group, the difference failed to reach the level of statistical significance (*P *= 0.130).

There was no significant difference in the frequency of nevus and angiomas between the two groups. However, lentigines were observed in five PEXG patients, while none of the POAG patients had this finding (*P *= 0.013).

In the subgroup analysis, there was no association between gender or patients' age and the frequency of dermatologic manifestations (*P* > 0.154 for all comparisons). Moreover, the frequency of dermatologic findings was not correlated with VA, IOP, or CDR (*P* > 0.142 for all comparisons).

##  DISCUSSION

In this cross-sectional study, we reported the frequency of skin manifestations in patients with PEXG and POAG. Interestingly, all included PEXG patients and most (92.3%) POAG subjects had at least one dermatologic finding.

Glaucoma is the most common cause of preventable, irreversible blindness in the world.^[16]^ With a prevalence of 1–5%, it was estimated that almost 80 million people would be affected by glaucoma in 2020.^[17]^ Considering the prevalence of the disease and its significant health impact, investigation of the full range of clinical manifestations of the disease and its comorbidities, including skin manifestations, seems to be highly relevant.^[18]^

While POAG is the most common form of the disease, PEXG is the most common secondary type of glaucoma.^[19]^ PEXS is a multi-organ systemic disease characterized by generalized microfibrillopathy.^[20]^ A mutation in the *lysyl oxidase-like-1 (LOXL1)* gene can be a culprit initial event;^[21]^ however, different environmental factors, including biological stress and free radicals, infectious agents, and trauma, can initiate the disease process.^[22,23,24,25]^ In addition to glaucoma, PEXS has a wide range of systemic comorbidities, including but not limited to cardiovascular disease, cerebrovascular disease, Alzheimer's disease, and sensorineural hearing loss.^[26,27,28,29]^ Moreover, precipitation of pseudoexfoliative materials has been shown in different organ systems, including viscera, blood vessels, skin, and ocular tissues.^[1,30]^

Pseudoexfoliative material is a highly cross-linked glycoprotein-proteoglycan complex, mainly composed of elastic microfibrillar components, such as fibrillin-1 and latent transforming growth factor binding proteins (LTBP), as well as chaperone molecules, such as clusterin, and cross-linking enzymes, such as LOXL1 protein.^[31]^ In some reports, these materials were shown to possess amyloid or amyloid-like features.^[32]^ In 1993, Schlötzer-Schrehardt and colleagues examined eyelid skin biopsy specimens of 12 PEXS patients. They found pseudoexfoliative material accumulations in the specimen of seven patients, which was confirmed by electron microscopy and immunological markers.^[33]^ Their findings are in line with other reports that found pseudoexfoliative materials in extraocular structures such as rectus and oblique muscles, vortex vein, optic nerve sheath, and the skin of the lateral canthus.^[34]^Their findings provide first-hand data on dermatological involvement in PEXS. Despite these clinicopathological correlations, there is no published study on clinical dermatological findings in PEXG patients. Through the current research we are reporting a broad group of skin disorders in these patients; our findings can further support the possible association between PEXS and skin disorders.

We observed a high prevalence of skin manifestations in all glaucoma patients in the present study. The most common dermatological manifestations in both groups were seborrheic dermatitis, dry skin, and cherry angioma. Erb et al reported skin diseases, including dry skin, as one of the most common comorbidities seen in all glaucoma patients.^[13]^ Their results are in line with our findings; however, they did not describe multiple types of skin lesions in their study. In addition, skin reactions were described in association with the use of anti-glaucoma eye drops.^[14]^ There was no significant difference between our study groups in terms of glaucoma severity and management; however, we excluded patients with apparent allergic dermatitis from topical glaucoma medication, as investigating the possible role of topical medications was beyond the scope of this study with its cross-sectional design.

Seborrheic dermatitis was the most common dermatological finding in both PEXG and POAG patients. Several studies reported a prevalence of 1–15% for seborrheic dermatitis in the general population,^[35,36,37]^ which is much lower than the observed frequency (57.1–61.5%) in our patients. This difference in prevalence can be either due to an association between the seborrheic dermatitis and glaucoma or the presence of dermatological issues due to lower hygiene and skincare in glaucoma patients. There is evidence that patients with advanced glaucoma may have poorer socioeconomic conditions than those with mild glaucoma.^[38]^ As a counterhypothesis, glaucoma may be secondary to primary dermatological disease. Of note, glaucoma has also been reported following the use of topical corticosteroids to treat eyelid dermatitis, such as seborrheic and atopic dermatitis.^[39]^ The eyelid's chronic inflammatory processes like seborrheic dermatitis and meibomian gland dysfunction may also lead to glaucoma. It has been proposed that regular lid hygiene could prevent or treat glaucoma.^[40]^

Our study had several limitations. As this was a cross-sectional study, we could only report on possible associations, and no causal relationship could be speculated based on our findings. Moreover, we had a limited sample size. Indeed, the lack of statistical significance in many comparisons could be merely because of insufficient statistical power. For example, twice higher frequency of dry skin in PEXG patients than in POAG patients did not reach statistical significance; on the other hand, although PEXG patients were almost a decade older than POAG patients, we did not find any effect of age on the frequency of skin manifestations. One can acceptably argue that the higher frequency of dry skin in PEXG was because they were older, and we 'failed' to demonstrate the effect of age on this association. However, in the subgroup analysis, we had the same number of elderly subjects in both groups, which can decrease the effect of age on the observed difference in the frequency of dry skin. Finally, we did not have a healthy control group with matched age, sex, and socioeconomic status, which was a major study limitation. Presence of that group could further help to clarify the possible association of dermatological findings with glaucoma versus other confounding factors like age, job, and socioeconomic condition.

Despite these limitations, to the best of our knowledge, the current study is the first one that investigated the dermatologic manifestations in glaucoma patients. Future studies with larger sample sizes, more diverse glaucoma patients, and a healthy control group can shed light on this less investigated aspect of glaucoma.

In summary, we noticed a wide range of dermatologic findings in POAG and PEXG patients. We recommend ophthalmologists and dermatologists to collaborate by referring these types of patients to each other for more comprehensive evaluation and management of such possible comorbidies. This can help diagnose and treat glaucoma patients earlier and shift the focus from a merely ocular condition to a multi-system disorder.

##  Financial Support and Sponsorship 

The study was supported by a research grant (No. 941297) from the Vice-Chancellor of Research at Mashhad University of Medical Sciences.

##  Conflicts of Interest

The authors declare no potential conflicts of interest for this article's research, authorship, and publication.
